# Single-Stage Primary Palatal Closure and Bone Grafting: A Comparison Between Unilateral Cleft Lip and Alveolus and Unilateral Cleft Lip and Palate

**DOI:** 10.7759/cureus.83347

**Published:** 2025-05-02

**Authors:** Hideto Imura, Teruyuki Niimi, Maya Yoshida, Chisato Sakuma, Masaaki Ito, Yasunori Akiyama, Ken Kitagawa, Nagana Natsume, Hiroo Furukawa, Nagato Natsume

**Affiliations:** 1 Cleft Lip and Palate Center, Aichi Gakuin University, Nagoya, JPN; 2 Division of Research and Treatment for Oral and Maxillofacial Congenital Anomalies, School of Dentistry, Aichi Gakuin University, Nagoya, JPN

**Keywords:** alveolar bone grafting, alveoloplasty, bone transplantation, cleft lip, cleft palate

## Abstract

Background

This study aimed to evaluate the outcomes of secondary alveolar bone grafting by assessing grafted bone resorption in the alveolar region and the anterior third of the hard palate in patients with unilateral cleft lip and palate (UCLP). A modified protocol involving simultaneous alveoloplasty and anterior palate closure was employed to optimize surgical outcomes and reduce the number of required procedures.

Materials and methods

A total of 55 patients were retrospectively evaluated: 16 with unilateral cleft lip and alveolus (UCLA) who underwent bone grafting limited to the alveolar cleft and 39 with UCLP who received simultaneous alveolar bone grafting and closure of the anterior one-third of the hard palate. All patients underwent surgery between 2012 and 2019. Surgical outcomes were assessed using the 6-point Standardized Way to Assess Grafts (SWAG) scale, based on postoperative dental radiographs. Bone fill was independently evaluated at the coronal, middle, and apical thirds of the cleft site.

Results

The median ages at surgery were 10.5 years (range: 8.75-12.6) for the UCLA group and 10.59 years (range: 8.33-15.3) for the UCLP group, with no significant age difference between groups. The mean total SWAG scores indicated favorable bone graft outcomes in both. Notably, complete bone fill (score 2) in the apical third was observed in all patients. No significant difference was found in bone resorption patterns between groups, and no resorption was noted on the apical side in either group. The difference in bone level at each third (coronal, middle, apical) between groups was not statistically significant. These findings highlight the efficacy of simultaneous anterior palate closure in reducing graft resorption and enhancing bone stability.

Conclusions

Simultaneous alveolar bone grafting and anterior hard palate closure in patients with UCLP is a clinically effective treatment protocol. It achieves stable bone regeneration with minimal resorption, reduces the need for multiple procedures, and enables reliable assessment through routine clinical radiographs. The SWAG scale proved to be a practical and reproducible tool for postoperative evaluation, and these findings provide valuable baseline data for clinical decision-making and informed consent.

## Introduction

Cleft lip and palate is a congenital anomaly that occurs in 1 out of 500-600 people in Japan. The pathology of cleft lip and palate is a lack of continuity of the lips, alveolar region, and palate. Orthodontic treatment is inadequate to treat an alveolar cleft because of the bony defects in the alveolar bone. Therefore, bone grafting enhances the effectiveness of cleft lip and palate treatment.

In 1972, Boyne and Sands demonstrated that fresh autogenous particulate cancellous bone and marrow (PCBM) graft to the alveolar cleft site allows eruption induction and orthodontic movement of cleft-adjacent teeth, occlusal morphology, and functional recovery without prosthetic treatment [1]. Many reports have described that clinical outcomes of alveolar cleft bone grafts are assessed with the Bergland scale [2], Kindelan scale [3], Chelsea scale [4], and Enemark score [5] using 2D radiographic images.

Several recent studies on alveolar clefts have used CT imaging for the 3D measurement of alveolar cleft volume to assess the operative outcomes of secondary alveolar bone grafting (SBG). In some studies, the preoperative alveolar cleft volume was measured to study the correlations with bone graft volume. Furthermore, the engraftment of the grafted bone was assessed with a focus on the postoperative changes to the cleft volume [6-13].

SBG is an important method for alveoloplasty and the recovery of occlusal function. Preoperative orthodontic treatment and timing of surgery are usually determined through consultation between orthodontists and oral surgeons.

Russell et al. considered the need for a bone graft scale that can be implemented at various developmental stages following bone grafting [14,15]. Therefore, to accommodate this need, the Standardized Way to Assess Grafts (SWAG) scale was developed, whereby analysis can be conducted without excessively detailed and complex measurements or methodology. The SWAG scale can assess both mixed and permanent dentition using routine clinical records, such as root apex and occlusion radiography. A higher score is considered indicative of a higher success rate of the graft. The SWAG scale is used for dividing the alveolar cleft-adjacent tooth root into thirds and scoring the bone volume of each third over 2 points, for a total score of 0-6 points, based on simple radiographs. Many recently established methods of bone graft assessment use CT and other imaging techniques; however, the SWAG scale, which enables simple assessment using routine clinical records, was deemed more effective in monitoring osteogenesis and engraftment.

In our hospital, the push-back technique is used in palatoplasty for unilateral cleft lip and palate (UCLP). This technique omits hard palatal closure at approximately age four to five years in the anterior one-third of the fistula area. Instead, a closure plate is used at the fistula site. In addition, a resin plate is attached to the orthodontic appliance to close the fistula site. After orthodontic treatment is initiated and the dentition is enlarged sufficiently, alveolar bone grafting and hard palate closure are performed at approximately 10 years of age in our hospital. The intervention protocol in our hospital has been hard palate closure and alveolar bone grafting for UCLP. Double-stage palatoplasty has several strong points; however, the increased number of surgical interventions is disadvantageous, prompting a major concern with this approach. Therefore, we optimized the treatment plan to simultaneously perform hard palate closure with alveolar bone grafting. Simultaneous completion of alveolar bone grafting and hard palate closure reduces the number of surgeries, leading to enhanced patient outcomes.

In addition, we believe that placing the grafted bone from the alveolar region to the anterior one-third of the hard palate contributes to preventing the narrowing of the maxillary dentition after bone grafting. At our hospital, we have been performing this surgical method for many years with favorable outcomes; however, it had not been formally evaluated until now. Therefore, this study aimed to evaluate the amount of resorption of grafted bone by performing alveoloplasty and hard palate closure simultaneously by placing a grafted bone in the alveolar region and in the anterior one-third of the hard palate. Since alveolar bone grafting is performed in UCLA cases, a comparison was made between UCLA and UCLP. The present study highlights the use of the SWAG scale [14,15] to assess the outcomes of SBG in unilateral cleft lip and alveolus (UCLA) and UCLP.

## Materials and methods

This study was approved by the Ethics Committee of the Oral Care Association (E220003) and conducted in accordance with the Declaration of Helsinki. Informed consent was obtained from the parents of the patients.

The study included all patients who underwent SBG performed by the same surgeon between 2012 and 2019 at a university dental hospital. A total of 16 patients with UCLA and 39 with UCLP were included. Patients were selected retrospectively, and no exclusion criteria were applied. UCLA was used as the control group due to its more favorable grafting conditions, including fewer and smaller fistulas compared to UCLP.

All patients underwent preoperative nasoalveolar molding for three to six months, followed by cheiloplasty using the modified Cronin method and palatoplasty with the push-back technique. Prior to the bone graft procedure, orthodontic expansion and alignment were performed. The surgeries were performed at a mean age of 10.5 years (range: 8.75-12.6) in the UCLA group and 10.59 years (range: 8.33-15.3) in the UCLP group. There was no significant difference in the timing of the surgery between the two groups. Patients with UCLA were assessed 4.81 months postoperatively, and those with UCLP were assessed 4.81 months postoperatively, with no significant difference between the two groups (Table 1).

**Table 1:** Samples and treatment protocols SBG, secondary alveolar bone grafting; UCLA, unilateral cleft lip and alveolus; UCLP, unilateral cleft lip and palate

Parameter	UCLA	UCLP
Sample size	16	39
Presurgical orthopedics	Yes	Yes
Cheiloplasty (average age)	Modified Cronin method at five months	Modified Cronin method at five months
Primary bone grafting	No	No
Palatoplasty	No	Pushback technique
Pre-SBG orthodontics	Expansion and alignment	Expansion and alignment
Timing of bone grafting (years)	Average: 10.5; range: 8.75-12.6	Average: 10.59; range: 8.33-15.3
Postoperative evaluation period (months)	Average: 4.81; range: 3-7	Average: 4.81; range: 3-9

The bone volumes for UCLA and UCLP were 4.55 g and 6.14 g, respectively. Furthermore, there was no residual fistula or infection for either UCLA or UCLP postoperatively.

The surgical technique followed the method of Bergland et al. [2]. On the vestibular side, the incision is made along the gingival border to ensure that the gingival flap contains the required width of attached gingiva. Posteriorly, the incision is extended to the first permanent molar, where it is angled up into the sulcus. To provide sufficient mobility, it is often necessary to cut through the periosteum at the base of the flap. Anteriorly, the incision is extended to the mesial aspect of the central incisor. Vertical incisions are made along the edges of the cleft. Wide exposure of the cleft area is achieved through these incisions. On the palatal side, mucoperiosteal flaps are raised along the edges of the cleft. The mucoperiosteum is lifted off the bone in the cleft region and off the frontal and lateral segments of the maxilla. All soft tissue is carefully stripped off the residual alveolar cleft. The nasal mucoperiosteum is pushed upwards, and if a fistula is present, the nasal floor is reconstructed. On the palatal side, mucoperiosteal flaps are raised along the edges of the cleft, and usually the margins are trimmed to fit together before suturing with everting mattress sutures. The alveolar cleft is now delimited by denuded bone medially and laterally and by the raw surface of mucoperiosteum on the nasal and palatal side. The gingival flap was raised to close the alveolar cleft site in the PCBM graft. The graft bed was formed to a sufficient height above the inferior border of the piriform aperture. The PCBM harvested from the iliac bone was packed to the height of the cement-enamel junction. Finally, the mucoperiosteal flap of the oral vestibule was released for closure.

Patients were evaluated using the SWAG scale (Table 2), which scores bone fill and root coverage in three segments (apical, middle, and coronal thirds) for a total of 6 points.

**Table 2:** SWAG scale scores SWAG, Standardized Way to Assess Grafts [14,15]

Score	Description
0	No bone bridge; permanent tooth roots are exposed within the cleft site.
1	No bone bridge; permanent tooth roots are not exposed within the cleft site.
2	Bone bridge is present in one-third of the cleft site (less than half of the total cleft filled); permanent tooth roots are exposed in both of the remaining unbridged thirds.
3	Bone bridge is present in one-third of the cleft site (less than half filled); permanent tooth roots are exposed in one of the remaining unbridged thirds but not in the other.
4	Bone bridge is present in one-third of the cleft site (less than half filled) with no root exposure in the remaining unbridged thirds, or bone bridge is present in two-thirds of the cleft site (more than half filled) with root exposure in the remaining unbridged third.
5	Bone bridge is present in two-thirds of the cleft site (more than half filled), with no permanent tooth root exposure in the remaining unbridged third.
6	Complete bone fill: clearly more than two-thirds of the cleft site is filled, including coverage up to or beyond the actual or projected root apices.

The SWAG scale is as follows: A score of 0 indicates that there is no bone bridge, and the permanent tooth roots are exposed in the cleft site. A score of 1 also indicates the absence of a bone bridge; however, in this case, the permanent tooth roots are not exposed in the cleft site. A score of 2 signifies the presence of a bone bridge in one-third of the cleft site, meaning that up to one-third but less than half of the cleft is filled with bone. In this situation, the permanent tooth roots remain exposed in the other two unbridged thirds. A score of 3 indicates that a bone bridge is present, filling at least one-third but less than half of the cleft site. In this case, the permanent tooth roots are exposed in one of the remaining unbridged thirds, while the other unbridged third has no root exposure. A score of 4 represents a bone bridge filling at least one-third but less than half of the cleft site, with no permanent tooth root exposure in the remaining unbridged thirds. Alternatively, it may indicate that a bone bridge is present in two-thirds of the cleft site, meaning that more than half of the cleft is filled, but with permanent tooth root exposure still present in the remaining unbridged third. A score of 5 denotes that a bone bridge is present in two-thirds of the cleft site, meaning that more than half of the cleft is filled, with no permanent tooth root exposure in the remaining unbridged third. Finally, a score of 6 signifies complete bone fill-in, where more than two-thirds of the cleft site is definitely filled, extending up to and beyond the actual or projected root apices.

**Figure 1:**
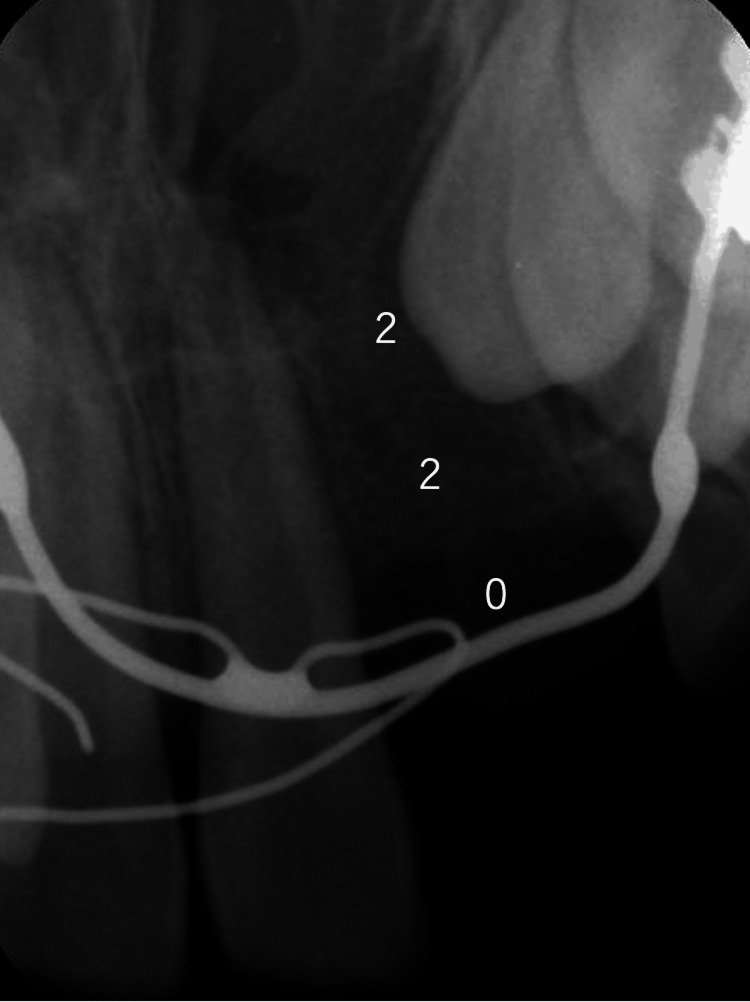
Measurement method of the SWAG scale Patients with less than one-third alveolar bone and gingival recession were assigned 0 points, those with less than one-third alveolar bone and no gingival recession were assigned 1 point, and those with preserved alveolar bone were assigned 2 points. The total score was 4 points. SWAG, Standardized Way to Assess Grafts

Based on simple clinical radiographs. Patients with less than one-third alveolar bone and gingival recession were assigned 0 points, those with less than one-third alveolar bone and no gingival recession were assigned 1 point, and those with preserved alveolar bone were assigned 2 points (Figure 1).

Patients were evaluated using the SWAG scale (Table 2), which scores bone fill and root coverage in three segments (apical, middle, and coronal thirds) for a total of 6 points. Additional evaluation was based on clinical radiographs, with alveolar bone height scored 0-2 points depending on the degree of bone preservation and gingival recession. The total possible score was 6 points for clinical bone height evaluation.

Two blinded observers independently assessed the radiographs. To assess intra-rater reliability, all radiographs were re-rated 48 hours later in random order. The score with the lowest intra-inspector error was adopted for each rater. Intra-rater reliability was 0.73 and 0.87, respectively; the rater with the lower error score was used in the analysis.

Statistical analysis was performed using IBM SPSS Statistics for Windows, Version 26.0 (Released 2019; IBM Corp., Armonk, NY, USA). Fisher’s exact test and chi-square test were used to compare differences between groups.

## Results

On the 6-point SWAG scale of patients with UCLA, 0% scored 1-3, 6.25% scored 4, 31.25% scored 5, and 62.5% scored 6. On the 6-point SWAG scale of patients with UCLP, 0% scored 1 or 2, 2.56% scored 3, 2.56% scored 4, 20.52% scored 5, and 74.36% scored 6 (Table 3).

**Table 3:** Distribution of the SWAG scores for UCLA and UCLP SWAG, Standardized Way to Assess Grafts; UCLA, unilateral cleft lip and alveolus; UCLP, unilateral cleft lip and palate

Score	UCLA (%) (N = 16)	UCLP (%) (N = 39)
Score 0	0	0
Score 1	0	0
Score 2	0	0
Score 3	0	2.56
Score 4	6.25	2.56
Score 5	31.25	20.52
Score 6	62.5	74.36

The distribution of SWAG scores in each third of the tooth root (coronal, middle, and apical) was evaluated for both the UCLA and UCLP groups (Table 4). For the coronal third, zero patients scored 0 points in either group. In the UCLA group, seven patients scored 1 point, and nine scored 2 points. In the UCLP group, nine patients scored 1 point, and 30 scored 2 points. There was no statistically significant difference between the groups (p = 0.191).

**Table 4:** Ratings of cleft site thirds for UCLA and UCLP UCLA, unilateral cleft lip and alveolus; UCLP, unilateral cleft lip and palate

Section	Group	Point 0	Point 1	Point 2	p-value
Coronal 1/3	UCLA	0	7	9	0.191
	UCLP	0	9	30	
Middle 1/3	UCLA	0	1	15	1.000
	UCLP	0	2	37	
Apical 1/3	UCLA	0	0	16	1.000
	UCLP	0	0	39	

For the middle third, no patients scored 0 points in either group. In the UCLA group, one patient scored 1 point, and 15 scored 2 points. In the UCLP group, two patients scored 1 point, and 37 scored 2 points. The difference between the groups was not statistically significant (p = 1.000).

For the apical third, all patients in both groups scored 2 points, indicating complete bone fill in that area. No significant difference was observed (p = 1.000).

## Discussion

Alveolar cleft bone grafting is a widely and commonly practiced procedure. However, the volume of the formed bone bridge remains a concern. In addition to conventional 2D assessment methods [2-5], recent studies report assessments using 3D measurements [6-13,16]. Rosenstein et al. studied assessments using CT and standard oral radiographs and found no significant difference between assessments using CT and 2D radiographs [17]. Although there are various methods of assessment, CT is used even by independent dental clinics today and is indispensable for detailed investigation of prognostic factors and accurate assessment of bone bridge formation. However, frequent CT examinations are considered unethical owing to radiation dose-related issues. Avoiding multiple CT scans and reducing radiation exposure in children is important.

Nightingale et al. [18] compared the assessment methods of Bergland et al. [2], Kindelan et al. [3], and Witherow et al. [4] and found their reliability to be comparable. Furthermore, there was no method with superior reproducibility for dental and occlusal radiographs.

Russell et al. stated that the SWAG scale uses routine clinical records, does not require extensive or expensive technology, provides sufﬁcient detail while being amenable to statistical analysis, and generates ratings that can be interpreted in the context of clinical treatment needs [14]. They also reported that the scores calculated with the SWAG scale after alveolar cleft bone graft in facilities were between 2.91 and 5.00 points.

In the present study, the presence or absence of a bone bridge and surgery prognosis were easily assessed. Regarding the eruption of teeth around the cleft, previous studies have reported that the eruption status of the induced tooth at the alveolar cleft site has no effect on the bone bridge rate [8,10,19]. In the present study, surgery was performed at approximately nine to 10 years of age, which is the time for canine eruption induction.

Post-bone grafting evaluations revealed that both UCLA and UCLP groups achieved a SWAG score of 2 for all patients in the apical one-third, indicating consistent and successful bone regeneration at the critical root apex level. This suggests that the timing of the surgery - performed around the eruption period of the permanent canines - was appropriate, and the surgical technique effectively supported bone stability in the deeper regions of the cleft. Although there was some variation in scores within the coronal one-third, the overall bone bridge formation was favorable, with no cases showing complete failure in bone regeneration. These favorable outcomes are likely attributed not only to the technical proficiency of the surgical approach but also to comprehensive perioperative oral care. Rigorous oral hygiene management before and after surgery likely contributed to the absence of postoperative infections, thereby promoting optimal healing conditions and enhancing graft success. Collectively, these findings support the validity and reliability of our surgical protocol and underscore the importance of a multidisciplinary treatment strategy that includes both surgical precision and proactive dental care.

The evaluation of bone level in the UCLP group showed results comparable to those of the UCLA group across all root regions. Notably, all patients in both groups scored 2 points for the apical third, indicating consistent and sufficient bone formation around the root apex, which is critical for long-term tooth stability and successful osteointegration.

Although a small variation was observed in the coronal third scores, especially in the UCLP group, none of the patients scored 0, and no cases exhibited apical resorption. This suggests that even when alveolar bone height was slightly reduced coronally, the overall bone graft was clinically successful. These findings emphasize that securing a stable scaffold and proper soft tissue management, such as sufficient periosteal release and flap tension reduction, is more influential to graft success than the original size or morphology of the cleft defect.

Given that both groups underwent bone grafting at a comparable age during canine eruption and that consistent surgical protocols, including simultaneous anterior fistula closure, were employed, the similarity in outcomes between the UCLA and UCLP groups validates the effectiveness of our timing and technique. Therefore, radiographic evaluation using the SWAG scale supports the clinical reliability and reproducibility of our treatment strategy for both simple and more complex cleft types.

We compared the results after bone grafting using radiographic findings and created baseline data useful for informed consent regarding alveolar bone grafting. Since Boyne and Sands first reported bone grafting in 1972 [1], the procedure has been refined to be performed before canine eruption, when the patient weighs more than 20 kg, and after orthodontic preparation to align the maxillary arch and reduce the cleft width. In our study, both the UCLA and UCLP groups achieved a mean SWAG score of 2.0 in the apical third, indicating that bone formation at the root apex level was highly successful. Compared to mean SWAG scores reported by four major cleft centers in North America and Europe (4.53, 2.9, 3.63, and 5.0) [14], the present findings demonstrate that our surgical protocol achieves favorable and clinically acceptable outcomes. These results can serve as valuable reference data when explaining surgical outcomes and expectations to patients and their families prior to surgery.

In recent years, methods for assessing the volume and quality of grafted bone using CT and other imaging modalities have been developed. However, the SWAG scale enables practical and ethical evaluation using standard dental radiographs, avoiding excessive radiation exposure - especially critical for pediatric patients. This approach allows for reliable monitoring of bone regeneration and graft integration while minimizing the burden on patients.

Nevertheless, this study has several limitations. It was conducted at a single institution, and all surgeries were performed by the same experienced surgeon. These factors may limit the generalizability of the findings to other clinical settings. Future multicenter studies with diverse operators are needed to further validate the reproducibility and clinical utility of our surgical protocol.

## Conclusions

This study evaluated SBG outcomes using the SWAG scale in patients with UCLA and UCLP. Our results demonstrated that simultaneous alveolar bone grafting with anterior hard palate fistula closure achieved high SWAG scores in both groups, with no significant differences observed in the coronal, middle, or apical thirds. Particularly, both groups showed complete bone fill (score 2) in the apical third, indicating excellent bone preservation. The use of a consistent surgical technique by a single surgeon, along with careful periosteal release and pre- or postoperative oral care, likely contributed to minimizing bone resorption.

The SWAG scale enabled simple, reproducible, and low-radiation evaluation using routine dental radiographs. Compared with international data from cleft centers, our outcomes were favorable and support the clinical validity of this protocol. This approach reduces the number of surgical interventions while achieving reliable graft stability and bone volume. Furthermore, the findings provide useful baseline data for informed consent discussions and long-term clinical planning in cleft care.
